# Hydrophobic Deep Eutectic Solvent-Derived Curcuminoids Loaded into a Double-Layer Colloidal Delivery System: Physicochemical Properties and In Vitro Biological Activity

**DOI:** 10.3390/foods15152601

**Published:** 2026-07-24

**Authors:** İrem Toprakçı, Mehmet Torun, Ebru Kurtulbaş, Mahmut Yildiztekin, Selin Şahin

**Affiliations:** 1 Department of Chemical Engineering, Istanbul University-Cerrahpaşa, Istanbul 34320, Türkiye; irem.toprakciyuksel@iuc.edu.tr (İ.T.); ebru.kurtulbas@iuc.edu.tr (E.K.); 2 Department of Food Engineering, Akdeniz University, Antalya 07070, Türkiye; torun@akdeniz.edu.tr; 3 Department of Crop and Animal Production, Vocational School of Köyceğiz, Muğla Sıtkı Koçman University, Muğla 48000, Türkiye; mahmutyildiztekin@mu.edu.tr

**Keywords:** turmeric, thymol–lactic acid system, Box–Behnken design, spray drying, MTT assay, cell viability

## Abstract

Hydrophobic deep eutectic solvents (HDESs) have been recognized as effective environmentally friendly extraction media for the recovery of lipophilic bioactive chemicals. Nonetheless, their incorporation with sophisticated delivery systems remains mostly unexamined. This study aimed to develop a curcuminoid-rich powder formulation from turmeric (*Curcuma longa* L.) and to evaluate its physicochemical properties, antioxidant activity, and effects on cell metabolic activity in vitro. Initially, 30 HDES systems based on thymol or menthol were prepared and compared in terms of their curcuminoid extraction performance. The thymol/lactic acid system (2:1, molar ratio) produced the highest total curcuminoid concentration among the screened HDES systems. The extraction parameters were subsequently optimized using a Box–Behnken response surface design. Under the optimized conditions, the total curcuminoid concentration in the analyzed extract solution was 6692.12 ppm. Then, the obtained extracts were incorporated into a double-layer emulsification system by high-pressure homogenization and converted into encapsulated powder form by spray-drying. The particle distribution index (PDI) of the prepared emulsion system was determined as 0.282, indicating a homogeneous distribution of particles within the emulsion system. The total curcuminoid-based encapsulation efficiency of turmeric powders obtained using a double-layer encapsulation system was determined to be 87.33%. Under separately simulated gastric and intestinal conditions, the detected curcuminoid recovery values were similar, at 31.24% and 31.10%, respectively. The ABTS radical-scavenging activity measured after the simulated intestinal treatment reached 81.57%. However, the possible contribution of residual HDES components to this activity could not be determined. The encapsulated powder caused a concentration-dependent reduction in the metabolic activity of DU-145 prostate cancer and Caco-2 colorectal adenocarcinoma cells in the MTT assay. To conclude, the integrated approach enabled the production of a curcuminoid-rich powder with high encapsulation efficiency. The findings support further investigation of the formulation’s technological and biological properties.

## 1. Introduction

Curcumin as the main bioactive component of turmeric (*Curcuma longa* L.) has significant potential in food, cosmetic, and pharmaceutical applications due to its antioxidant, anti-inflammatory, and antiproliferative properties [1,2]. Furthermore, its classification as a “Generally Recognized as Safe” (GRAS) ingredient by the US Food and Drug Administration (FDA) supports its use in functional food formulations [3]. However, curcumin’s low water solubility, chemical instability, and limited bioavailability in the gastrointestinal system significantly limit its application areas [4]. Therefore, in recent years, research has accelerated towards developing carrier systems to improve curcumin’s solubility, stability, and accessibility in the gastrointestinal system [5,6].

Emulsion-based encapsulation systems are among the effective approaches for the protection, controlled release and more efficient transport of lipophilic bioactive compounds in the gastrointestinal system [5,6]. Emulsion systems created by high-pressure homogenization are noteworthy for their advantages in the transport of functional components such as low energy consumption, high scalability and lack of need for organic solvents [7,8]. The incorporation of suitable biopolymers into emulsion systems has been reported to protect bioactive compounds against environmental conditions and modify their release behavior in gastrointestinal models [6].

On the other hand, there is increasing interest in environmentally friendly and sustainable solvent systems for the extraction of naturally occurring bioactive substances. Deep eutectic solvents (DESs) stand out as alternatives to traditional organic solvents due to their properties such as low toxicity, biodegradability, low volatility and high dissolution capacity [2]. Hydrophobic deep eutectic solvents (HDES) offer significant advantages in the extraction of natural compounds with low water solubility [9]. HDES systems consisting of terpene-based hydrogen bond acceptors such as menthol and thymol, and organic acids have been reported to increase the solubility of lipophilic compounds such as curcumin and have potential in terms of biological activity [10,11,12]. In addition, the biocompatibility of these solvent systems increases their potential for use in food and pharmaceutical applications.

Although numerous studies exist in the literature on the extraction, encapsulation, and biological activity of curcumin, studies investigating HDES extraction and bilayer microencapsulation systems together are quite limited [10,11,12,13]. In particular, there is a lack of comprehensive studies integrating environmentally friendly extraction approaches with emulsion-based carrier systems.

In this study, 30 thymol- and menthol-based HDES systems containing different organic acids were systematically screened for the extraction of curcuminoids from turmeric, and the extraction conditions of the selected thymol/lactic acid system were optimized using a Box–Behnken response surface design. The resulting extract was incorporated into a whey protein isolate–maltodextrin double-layer delivery system using high-pressure homogenization and subsequently converted into powder form by spray-drying. The physicochemical properties and antioxidant activity of the powder were characterized, its behavior under separately simulated gastric and intestinal conditions was evaluated, and its concentration-dependent effects on the metabolic activity of DU-145 and Caco-2 cells were investigated using the MTT assay. The novelty of this study lies in the integration of systematic HDES screening and extraction optimization with double-layer encapsulation and spray-drying within a single workflow for producing a curcuminoid-rich powder. This integrated approach provides information on both the technological properties and preliminary in vitro biological behavior of the resulting formulation.

## 2. Materials and Methods

### 2.1. Materials

Samples of turmeric (*Curcuma longa* L.) in dry and crumbled form were obtained from a local herbalist operating in Istanbul. Menthol (≥99%), thymol (≥99%), oleic acid (≥99%), capric acid (≥98%), caprylic acid (≥98%), pyruvic acid (≥98%), lactic acid (85%), curcumin standard (≥98%), Tween 80 (polysorbate 80; a non-ionic surfactant), maltodextrin, modified starch, Folin–Ciocalteu reagent, methanol (HPLC grade), acetonitrile (HPLC grade), and dimethyl sulfoxide (DMSO) used in the study were obtained from Sigma-Aldrich (St. Louis, MO, USA).

### 2.2. Preparation of Candidate Hydrophobic Deep Eutectic Solvents

HDESs were prepared using menthol and thymol as hydrogen bond acceptors (HBA) and oleic acid, capric acid, caprylic acid, pyruvic acid, and lactic acid as hydrogen bond donors (HBD) (Table S1). HDES systems prepared at different molar ratios were stirred using a magnetic stirrer at 80 °C until a clear appearance was obtained [14]. The prepared solvent systems were used in extraction studies.

### 2.3. Physicochemical Characterization of Hydrophobic Deep Eutectic Solvents

The prepared mixtures were characterized in terms of viscosity, density, and apparent pH. Viscosity was measured using a rotational viscometer (Anton Paar ViscoQC-300L, Graz, Austria), while density was determined at 25 °C using a pycnometer. Apparent pH values were measured directly using a pH meter (Mettler-Toledo GmbH, Schwerzenbach, Switzerland). The compositions, molar ratios, and measured physicochemical properties are provided in Tables S1 and S2. Since the systems were predominantly non-aqueous, the measured pH values are reported as apparent pH and should be interpreted only as comparative values among the prepared mixtures.

### 2.4. Extraction Procedure and Experimental Design

Turmeric samples were extracted using an automated solvent extraction system (SER 148/6, VELP Scientifica, Usmate, Italy). For each experimental run, the specified amount of dried turmeric sample was weighed and placed in a single-thickness cellulose extraction thimble (33 mm × 80 mm; Whatman, Maidstone, UK). The extraction thimble was then placed in a glass extraction vessel containing 80 mL of HDES.

The automated extraction cycle consisted of immersion, removal, washing, solvent recovery, and cooling stages. During the immersion stage, the thimble containing the turmeric sample was immersed in the heated extraction medium for the time specified in the experimental design. Subsequently, the solvent level was automatically lowered below the extraction thimble. During the washing stage, the extraction medium was allowed to pass through the sample matrix for the specified period. The solvent recovery stage was then initiated. Finally, the heating system was switched off, and the glass vessels containing the extracts were automatically raised for cooling. The resulting extracts were collected for subsequent analyses.

A three-factor, three-level Box–Behnken design comprising 17 experimental runs was applied to evaluate the effects of solid mass (A; 0.5–1.5 g), immersion time (B; 10–30 min), and washing time (C; 20–40 min) on the total curcuminoid concentration in the analyzed extract solution. The factor ranges were selected to evaluate the effects of solid mass, immersion time, and washing time within experimentally feasible operating conditions. Because the highest predicted response occurred at or near the upper limit of washing time, the investigated range may not have fully encompassed the response maximum for this factor. Experimental design, regression analysis, analysis of variance, and numerical optimization were performed using Design-Expert software (version 12, Stat-Ease Inc., Minneapolis, MN, USA).

### 2.5. Preparation of Double-Layer Microencapsulated System

Total curcuminoid-rich extracts obtained under optimum extraction conditions were encapsulated using a double-layer microencapsulation system. Whey protein isolate was used as the primary coating material, while maltodextrin was used as the secondary coating material. Tween 80, used as a non-ionic surfactant, was added at a concentration of 0.1% (*w*/*v*) to a 30% (*w*/*v*) whey protein isolate solution. Turmeric extract was added dropwise to the solution under continuous stirring. The resulting primary emulsion was added to a 30% (*w*/*v*) maltodextrin solution to create a double-layer coarse emulsion system.

The prepared coarse emulsion was processed in a high-pressure homogenizer at 500 bar pressure. The resulting emulsions were converted into microencapsulated powder form using a laboratory-type spray-dryer (BUCHI B-290, Flawil, Switzerland). The drying process was carried out at an inlet temperature of 160 °C, an outlet temperature of 85 ± 5 °C, and an air flow rate of 550 L/h. The resulting powder samples were stored at −18 °C until analysis.

### 2.6. Characterization of Encapsulated Powders

Zeta potential, particle size, and polydispersity index (PDI) values of emulsion systems were determined using dynamic light scattering spectrometry (DLS) (Anton Paar Litesizer 500, Graz, Austria) [15]. Moisture content of microencapsulated powders was measured using a moisture analyzer (MAC 50/NH, RADWAG, Radom, Poland) [16]. Additionally, water activity was measured using an AquaLab 4TE water activity meter (METER Group, Pullman, WA, USA) [17].

In bulk density (ρ_b_) analysis, a specific amount of powder sample was carefully transferred into a calibrated 50 mL graduated cylinder, and the volume occupied by the particles was recorded. Bulk density was calculated by dividing the sample mass by the measured volume. The cylinder was struck 100 times against a fixed surface in a controlled manner to rearrange the particles in compressed density (ρ_t_) analysis. Later, the compressed density was calculated using the resulting final volume value [18].

The flowability properties of the powder samples were evaluated using the Carr index (CI) [19]. CI was calculated according to the following equation:

(1) $$CI=\frac{\rho_{t}-\rho_{b}}{\rho_{t}}\times 100$$

In the wettability analysis of microencapsulated powders, 0.1 g of sample was added to a beaker containing room temperature pure water using a funnel, dispersing it on the surface, and the time taken for the powder particles to be completely wetted was recorded in seconds.

In the solubility analysis, 1 g of powder sample was dispersed in 25 mL of pure water and mechanically mixed at 3000 rpm for 5 min. After centrifuging the resulting dispersion at 3000 rpm for 5 min, the supernatant was transferred to Petri dishes and dried at 105 °C for 4 h. The solubility value was calculated as a percentage (%) of the dry matter amount obtained after drying [19].

### 2.7. HPLC Analysis of Curcuminoids

The total curcuminoid content of turmeric extracts obtained by deep eutectic solvent extraction was determined by modifying the method reported by Wichitnithad et al. [20]. The method proposed by Takma et al. [21] for curcuminoid extraction was applied with some modifications. For total curcuminoid extraction, 15 mL of methanol:water (containing 0.1% HCl) (80:20, *v*/*v*) solution was added to 2.5 g of sample. The mixture was incubated in a water bath at 60 °C for 20 min and then centrifuged at 4000 rpm for 5 min. The resulting supernatant was filtered through a 0.45 µm syringe filter and used in HPLC analysis.

To determine the surface curcuminoid content of microencapsulated powder samples, 2.5 g of sample was mixed with 100% methanol, briefly vortexed, and then centrifuged at 4000 rpm for 5 min. The resulting supernatant was filtered through a 0.45 µm membrane filter and analyzed. Curcumin analyses were performed using an HPLC system consisting of degassing unit (DGU-20A5, Shimadzu Corporation, Kyoto, Japan), a pump unit (LC-20AD, Shimadzu Corporation, Kyoto, Japan), an automatic sampler (SIL-20AD, Shimadzu Corporation, Kyoto, Japan), a column oven (CTO-20AC, Shimadzu Corporation, Kyoto, Japan), and a diode-array detector (SPD-M20A, Shimadzu Corporation, Kyoto, Japan). Separation was performed using an Inertsil ODS-3 C18 column (GL Sciences Inc., Tokyo, Japan). A water mixture containing acetonitrile and 2% acetic acid (40:60, *v*/*v*) was used as the mobile phase. The flow rate was set at 2.0 mL/min, while the injection volume was set at 20 µL, and the detection was performed at 425 nm. The analysis time was 30 min. The concentrations of curcumin, demethoxycurcumin, and bisdemethoxycurcumin were calculated from their respective HPLC calibration curves. Total curcuminoid concentration was calculated as the sum of these three individual concentrations. The reported ppm values refer to the HPLC-determined concentrations in the final filtered analytical solutions submitted to the HPLC system and not to the curcuminoid content per gram of dry turmeric. No mass-normalized extraction yield was calculated from these values.

Finally, encapsulation efficiency (EE) was calculated based on the difference between the total curcuminoid amount and the surface curcuminoid amount:

(2) $$EE\left(\%\right)=\frac{total\;curcuminoid\;amount-surface\;curcuminoid\;amount}{total\;curcuminoid\;amount}\times 100$$

### 2.8. In Vitro Evaluation Under Simulated Gastric and Intestinal Conditions

The behavior of the microencapsulated turmeric extract powder under simulated gastric and intestinal conditions was evaluated using a modified version of the static in vitro evaluation under separately simulated gastric and intestinal conditions reported by Minekus et al. [22]. Simulated gastric digestion fluid (SGF) and simulated intestinal digestion fluid (SIF) were prepared under appropriate enzyme and pH conditions to represent the respective gastrointestinal environments. All digestion solutions were heated to 37 °C prior to analysis. The gastric and intestinal incubations were conducted independently using separate aliquots of the microencapsulated powder. Thus, the intestinal samples were not obtained by transferring the gastric digesta to the intestinal phase.

For gastric digestion, 2.5 g of microencapsulated sample was mixed with a SGF solution adjusted to pH 1.2. The SGF solution was prepared to contain pepsin, NaCl, and HCl, and the total volume was completed with distilled water. The mixtures were incubated at 37 °C and stirring speed of 100 rpm for 2 h. At the end of the digestion process, the reaction was stopped by the addition of ethanol. For small intestinal digestion, samples were mixed with an SIF solution containing pancreatin and KH_2_PO_4_ and adjusted to pH 6.8. The mixtures were incubated at 37 °C at 100 rpm for 2 h. The digestion process was terminated by the addition of ethanol. Post-digestion samples were centrifuged at 4000 rpm for 5 min, and the supernatant was passed through a 0.45 µm membrane filter to prepare it for HPLC analysis. Curcuminoid amounts were determined using an HPLC system as described above.

Total phenolic content of post-digestion samples was determined spectrophotometrically at 760 nm using the Folin–Ciocalteu method. Antioxidant activity values were evaluated using the ABTS (2,2′-azino-bis(3-ethylbenzothiazoline-6-sulfonic acid)) radical scavenging method, where the absorbance values were measured at 734 nm.

Since the gastric and intestinal treatments were conducted independently rather than sequentially, this method was used to compare the behavior of the powder under separately simulated gastric and intestinal conditions. It does not reproduce the complete sequential gastrointestinal digestion process.

### 2.9. Cell Culture and Antiproliferative Activity

Antiproliferative activity analyses were performed using DU145 human prostate cancer and Caco-2 human colorectal adenocarcinoma cell lines. Cells were cultured in DMEM medium supplemented with 10% fetal bovine serum (FBS), L-glutamine, and penicillin/streptomycin at 37 °C in a humidified atmosphere containing 5% CO_2_. The DU145 (ATCC HTB-81) and Caco-2 (ATCC HTB-37) cell lines were obtained from the American Type Culture Collection (ATCC, Manassas, VA, USA).

After seeding the cells into 96-well plates, microencapsulated samples were applied at different concentrations. Cell viability was determined using the MTT method, and absorbance values were measured using a UV/Vis spectrophotometer [23,24]. IC_50_ values were calculated using GraphPad Prism 8 (GraphPad Software Inc.; San Diego, CA, USA). Untreated cells maintained under the same experimental conditions were used as the negative control. The metabolic activity of the treated cells was expressed relative to that of the untreated cells. A pharmacological positive control was not included because the assay was designed as a preliminary evaluation of the concentration-dependent response to the encapsulated powder rather than as a comparative potency assessment against an established cytotoxic agent.

### 2.10. Statistical Analysis

The significance and interaction of independent variables were evaluated using ANOVA via the Design Expert software (Version 12.0.1.0, Stat-Ease Inc., Minneapolis, MN, USA). Additionally, one-way ANOVA was used to determine differences between HDES groups using Minitab Statistical Software 22 (Minitab Inc., State College, PA, USA). The Tukey test was used for post hoc pairwise comparisons. A significance level of *p* < 0.05 was used for all statistical analyses. 95% confidence intervals (α = 0.05) were calculated to determine the sensitivity of mean differences.

## 3. Results and Discussion

### 3.1. Screening and Selection of Hydrophobic Deep Eutectic Solvent Systems

In the first phase of the project, 30 thymol and menthol-based HDES samples were prepared. Table S1 shows the components and their molar ratios. Table S2 shows the viscosity, density, and pH values of the HDESs. According to the results obtained, viscosity values ranged from 11.10 to 57.46 mPa·s, while density values ranged from 0.6769 to 1.1894 g·cm^−3^. pH values were found to vary between 0.01 and 1.54. The formation of a homogeneous liquid at room temperature is consistent with the operational behavior commonly reported for terpene–organic acid eutectic mixtures. In such systems, hydrogen-bond interactions between the terpene and organic-acid components may contribute to melting-point depression and stabilization of the liquid state. Previous studies have similarly reported that the physicochemical properties of thymol- and menthol-based mixtures depend strongly on the identities and molar ratios of their components [25,26].

To determine the curcumin content of the samples, a curcumin standard (AFG Bioscience 273571 Curcumin) dissolved in ethanol was injected into the HPLC system. The chromatogram obtained as a result of the analysis is given in Figure S1. When the chromatogram appearance was examined, it was determined that the curcumin standard contained different curcuminoids. In the study conducted by Wichitnithad et al., it was reported that the samples contained curcumin, demethoxycurcumin and bisdemethoxycurcumin as a result of chromatographic analysis of turmeric extracts [20]. The presence of curcumin (15.87), demethoxycurcumin (14.87), and bisdemethoxycurcumin (13.91) curcuminoids were detected in the structure of the standard used in the study, showing similarity with the literature. Calibration curves prepared to determine the amount of curcuminoids are given in Figure S2, while the comparison of the sample and the spike chromatogram by adding the standard solution to the sample is given in Figure S3.

The individual and total curcuminoid concentrations determined in the analyzed HDES-based turmeric extract solutions are presented in Table 1. One-way analysis of variance (ANOVA) was also applied to the groups. A statistically significant difference was observed among the HDES systems in terms of total curcuminoid concentration (*p* < 0.05). According to Tukey’s multiple-comparison test, the highest total curcuminoid concentrations were obtained in Experiments 30 (6349.60 ppm), 28 (6130.36 ppm), and 29 (5844.40 ppm), whereas the lowest concentration was obtained in Experiment 26 (2086.94 ppm). These findings demonstrate that HDES composition significantly affected the total curcuminoid concentration measured in the analyzed extract solutions. To maintain the readability of Table 1, compact-letter groupings for all 30 formulations were not displayed.

It was deemed appropriate to continue the extraction studies with the thymol/lactic acid (2/1) system, since it provided the highest yield. This coincides with the relatively low viscosity and higher density of the solvent system compared to others. On the other hand, the observed behavior of the thymol/lactic acid system might be associated with eutectic-like behavior reported for similar terpene–organic acid systems, which has been described as contributing to liquid-state formation at room temperature. However, no thermal characterization (such as DSC) was performed in the present study to confirm eutectic formation. This property is also beneficial for processes where low temperature conditions are desired. Liu et al., highlighting the extractive properties of eutectic solvents and their usefulness in isolating valuable biocompounds, stated that the unique hydrogen bonding properties provided by the thymol/lactic acid system significantly increased the extraction efficiency [27]. This result is consistent with studies in the literature on the high dissolving capacity and reported solubilization properties of thymol-based eutectic solvents. Bagovi’c et al. also characterized 7 menthol and thymol-based eutectic solvents [28]. Thymol-based HDESs showed slightly higher polarity and density values than menthol-based ones. However, the opposite trend was observed for viscosity values. Furthermore, their antioxidant capacity and antimicrobial activity were determined, where thymol-based HDESs containing coumarin as a hydrogen bond acceptor and possessing the highest Oxygen Radical Absorbance Capacity (ORAC) value were found to be more potent as potential antioxidants.

The safety of the selected thymol/lactic acid mixture was not experimentally evaluated in the present study. Bagović Kolić et al. [28] reported no cytotoxic effects up to 1000 mg/L for seven other menthol- and thymol-based HDES formulations in three human cell lines. However, the biological properties of a deep eutectic mixture cannot be reliably predicted from its individual components or extrapolated from compositionally different HDES systems. Their findings therefore provide only general context and cannot be considered direct evidence for the safety of the thymol/lactic acid mixture investigated here. Furthermore, thymol and lactic acid residues were not quantified in the final encapsulated powder. Consequently, neither exposure to residual HDES components nor their contribution to the antioxidant and cell-based responses could be determined. Safety for functional-food or supplement applications cannot be inferred from the present data and would require residue quantification, toxicological assessment of the selected mixture, and evaluation of the final formulation using relevant non-cancerous cell and in vivo models.

### 3.2. Optimization of Extraction Parameters Using Response Surface Methodology

The extraction process using the selected HDES system was further optimized using response surface methodology with a Box–Behnken design. The effects of solid mass (A), immersion time (B), and washing time (C) on the total curcuminoid concentration in the analyzed extract solution ($Y_{TC}$) were investigated using the automated solvent extraction system (Table 2). The highest experimental response, 6534.19 ppm, was obtained in Run 15 using 1.5 g of turmeric, a 20 min immersion time, and a 40 min washing time. In contrast, the lowest response, 89.80 ppm, was obtained in Run 4 using 1.0 g of turmeric, a 10 min immersion time, and a 20 min washing time. These findings indicate that the investigated process variables affected the total curcuminoid concentration recovered in the analyzed extract solution.

As shown in Table 3, the overall quadratic model was statistically significant (*p* < 0.0001). The linear terms (A, B and C), the interaction terms (AB, AC and BC), and the quadratic terms (B^2^ and C^2^) were significant at *p* < 0.05. In contrast, the quadratic term A^2^ was not statistically significant (*p* = 0.1038). Nevertheless, A^2^ was retained in the model to preserve the hierarchical structure of the fitted quadratic equation. Among the investigated terms, washing time (C) had the highest F-value. This indicates that washing time exerted the strongest effect on the total curcuminoid concentration measured in the analyzed extract solution.

The experimental data were fitted to a second-order polynomial regression model generated by RSM analysis (Equation (3)):

(3) $$\begin{array}{c} Y_{\mathrm{TC}}\;=\;2181.86+1241.17\;A+699.55\;B+1572.46\;C+900.36\;AB+389.28\;AC\\ \;-602.98\;BC-179.79\;A^{2}-415.51\;B^{2}+1235.81\;C^{2} \end{array}$$

ANOVA results demonstrated that the developed model was highly significant (*p* < 0.0001) (Table 3). The lack-of-fit test is not significant (*p* > 0.05). Therefore, we can conclude that the model explains the experimental data well. The model’s R^2^ (0.9944) explains 99.44% of the variability, while the adjusted and estimated R^2^ (>0.94) values support the high explanatory and predictive power of the model. On the other hand, the coefficient of variation value (C.V.) as another evaluation criterion was calculated as 7.95%. This result shows that the variability between the values predicted by the model and the experimental data is very small. In conclusion, it supports the model being stable and reliable.

#### 3.2.1. Response Surface Analysis

This section presents response surface plots to visually evaluate the interactions of factors affecting the total curcuminoid concentration in the analyzed extract solution. Three-dimensional response surface plots show the combined effect of two independent variables on extract yield, while the other variable is kept constant. This allows for a clearer understanding of the interactions between factors and the optimum operating conditions.

**Figure 1:**
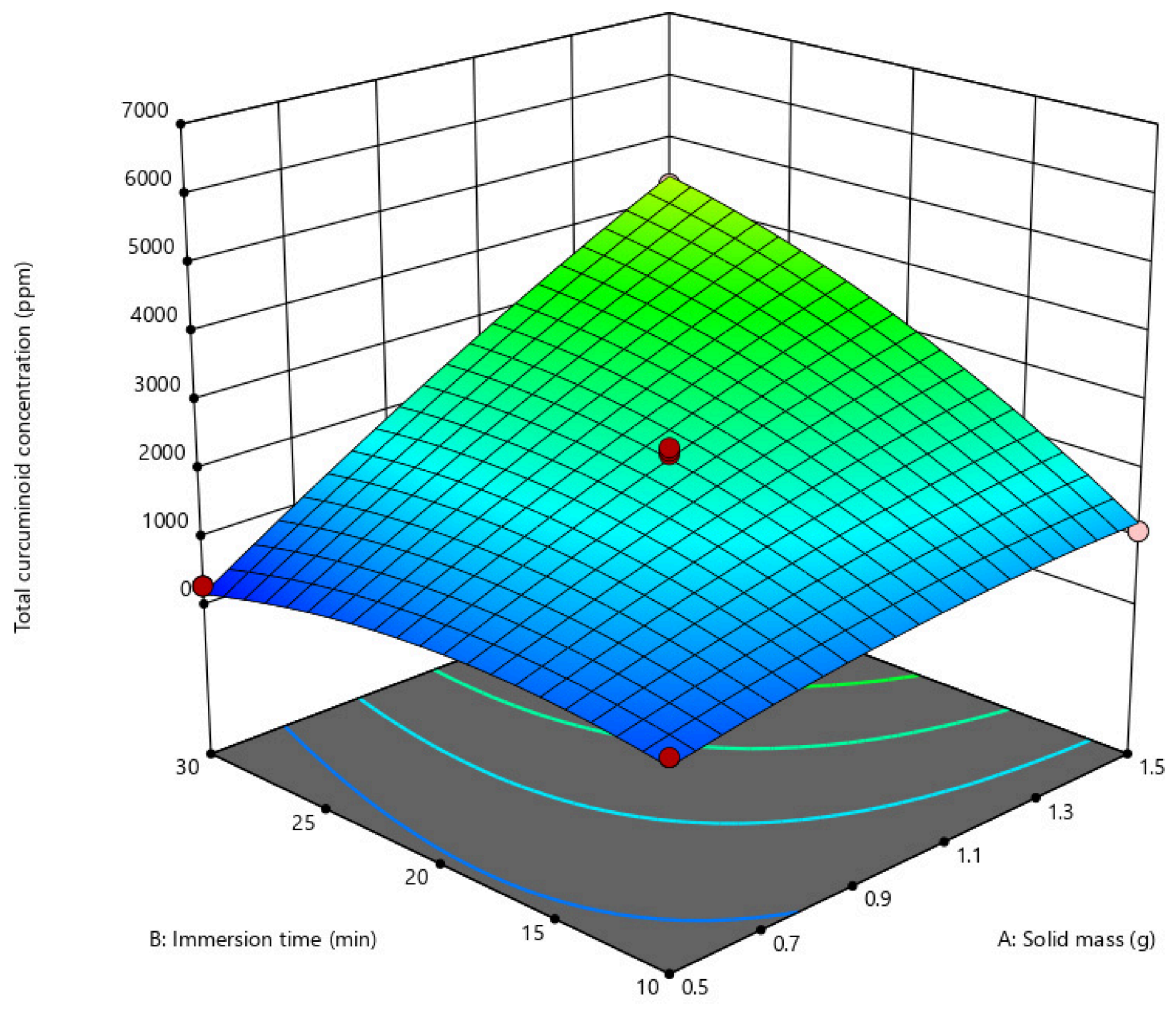
Three-dimensional response surface plot showing the interactive effects of solid mass and immersion time on total curcuminoid concentration in the analyzed extract solution, with washing time fixed at 30 min. The red circles represent the experimental data points, while the colored surface and contour lines represent the model-predicted response.

Examining the response surface plots, it is observed that increasing both solid matter amount and immersion time significantly increases the extract yield (Figure 1). This behavior may be attributed to enhanced mass transfer between the turmeric matrix and the HDES phase during prolonged immersion. Longer immersion times favor deeper penetration of the thymol/lactic acid solvent system into plant tissues, promoting the dissolution of curcuminoids through hydrogen-bond interactions and hydrophobic affinity. Similar observations have been reported for terpene-based HDESs used in the extraction of lipophilic phytochemicals, where increased contact time improved extraction efficiency and solubilization capacity [27].

**Figure 2:**
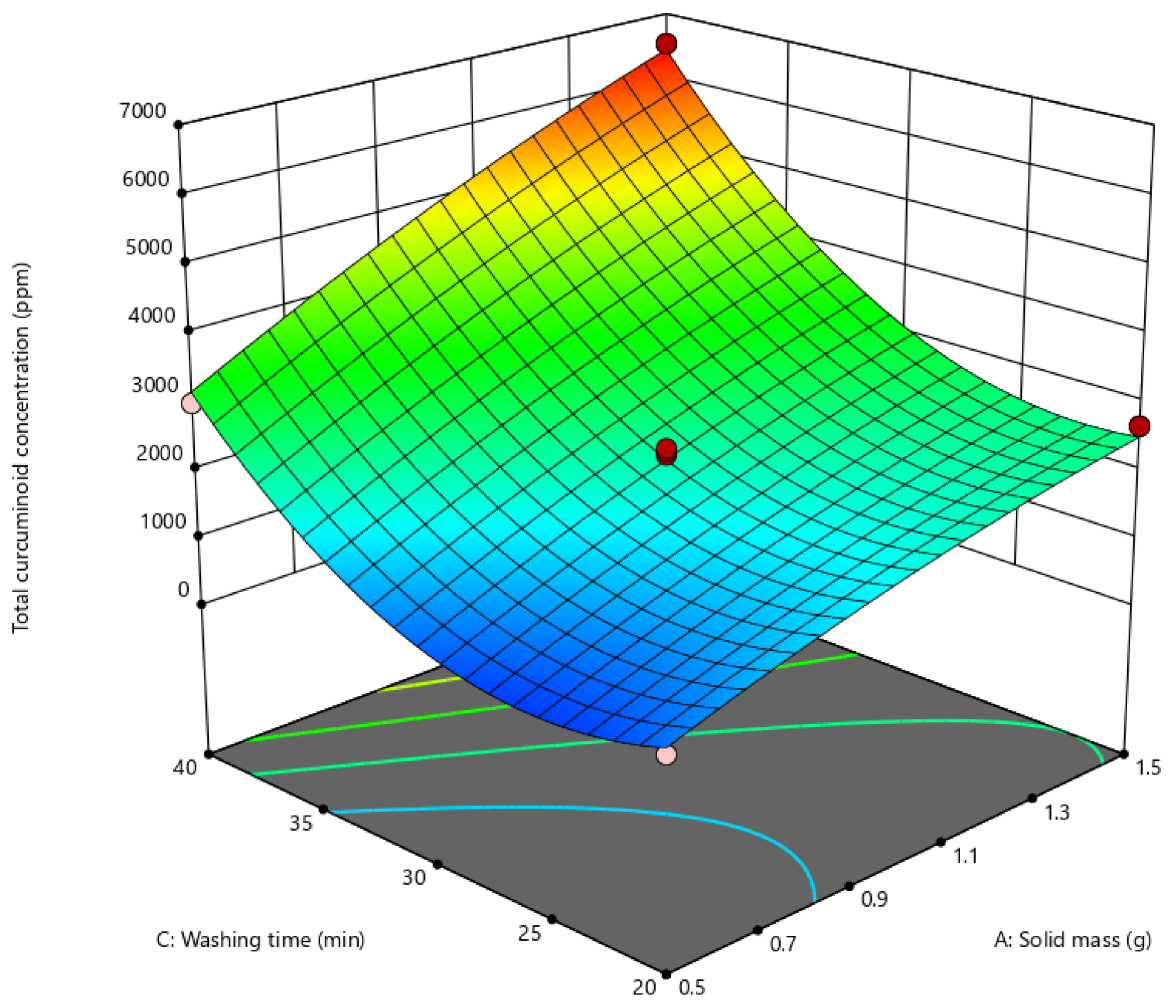
Three-dimensional response surface plot showing the interactive effects of solid mass and washing time on total curcuminoid concentration in the analyzed extract solution, with immersion time fixed at 20 min. The red circles represent the experimental data points, while the colored surface and contour lines represent the model-predicted response.

When the amount of solid matter and the washing time were evaluated together, it was determined that the optimum extract yield was obtained under conditions of approximately 1.4–1.5 g of solid matter and a washing time of 38–40 min (Figure 2). The positive effect of washing time may be associated with enhanced diffusion of curcuminoids from the plant matrix into the solvent phase. In addition, the relatively low viscosity and strong hydrogen-bonding capacity of the thymol/lactic acid (2:1) HDES likely improved solvent accessibility and facilitated curcuminoid release. Similar findings were reported by Liu et al. [27], who demonstrated that thymol-based eutectic solvents significantly enhanced the extraction efficiency of phenolic bioactive compounds due to their unique intermolecular interaction mechanisms.

**Figure 3:**
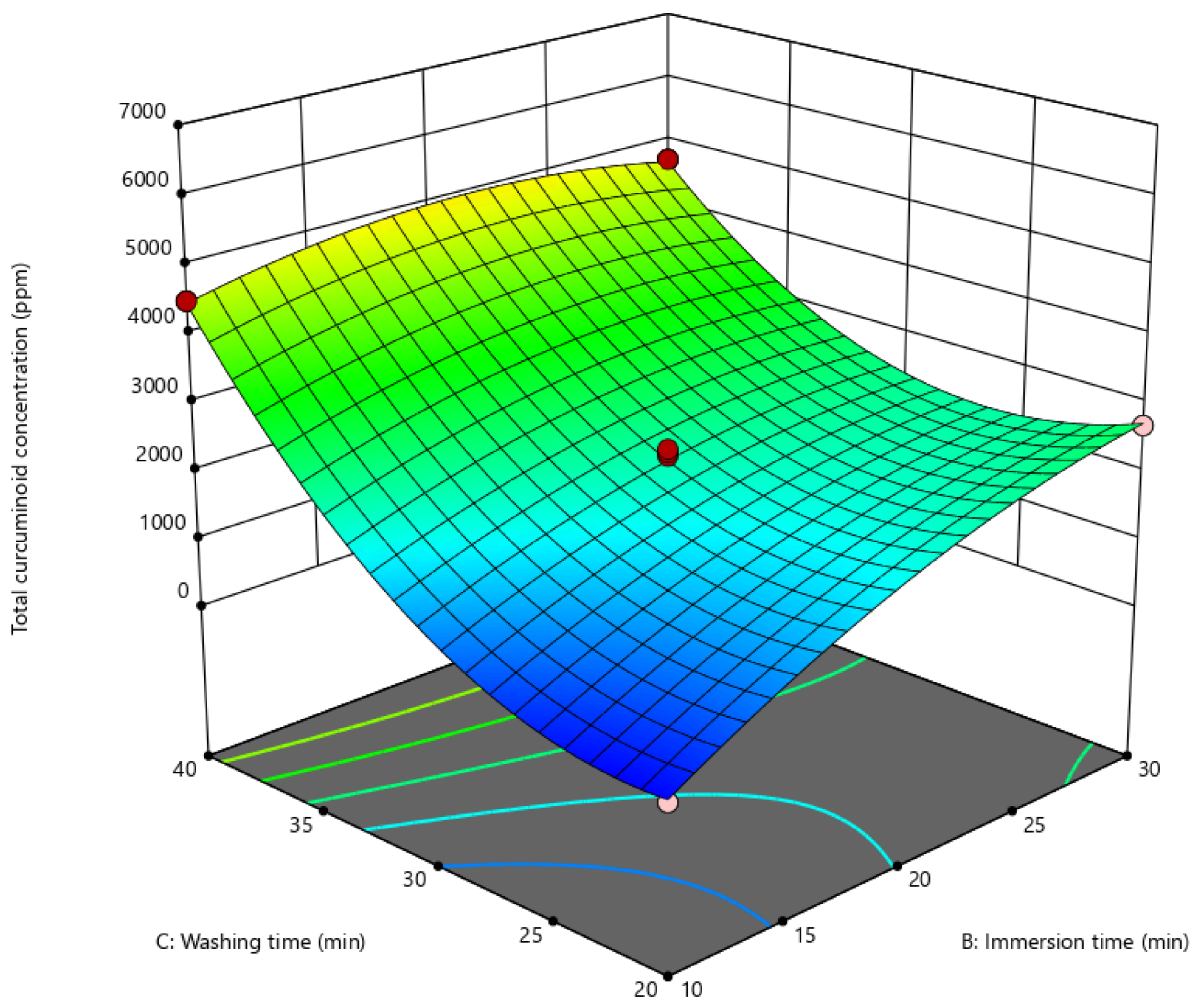
Three-dimensional response surface plot showing the interactive effects of immersion time and washing time on total curcuminoid concentration in the analyzed extract solution, with solid mass fixed at 1 g. The red circles represent the experimental data points, while the colored surface and contour lines represent the model-predicted response.

Furthermore, it was observed that increasing both the immersion time and the washing time positively affected the extract yield (Figure 3). This suggests that these variables synergistically improve solvent–solid interactions and mass transfer kinetics. The curved surface profile also indicates the presence of an optimum extraction region, which is characteristic of response surface methodology models applied to bioactive compound extraction processes. Previous studies have emphasized that thymol-based HDES systems possess high solubilization capacity toward hydrophobic compounds because of their amphiphilic nature, low volatility, and extensive hydrogen-bond network [1,2,7].

#### 3.2.2. Multi-Factor Optimization of Curcumin Extraction Using Thymol/Lactic Acid HDES

Within the investigated experimental domain, the conditions predicted to provide the highest total curcuminoid concentration were 1.465 g of solid mass, 25 min immersion time and 40 min washing time. Under these conditions, the model predicted a total curcuminoid concentration of 6564.18 ppm (Figure S4). The fitted quadratic model showed a high coefficient of determination (R^2^ = 0.9944), while numerical optimization yielded a desirability value of 1.00. The predicted washing time was located at, or close to, the upper boundary of the investigated range. Therefore, this condition should be interpreted as the best-performing condition within the studied experimental domain rather than as a confirmed internal optimum. The model suggests that the response had not reached a clear maximum with respect to washing time within the selected range. Evaluating washing times beyond the current upper limit would be necessary to determine whether the response continues to increase, reaches a plateau, or subsequently decreases.

A validation experiment was conducted under the model-predicted conditions. The predicted total curcuminoid concentration was 6564.18 ppm, while the experimentally measured value was 6692.12 ppm. The absolute prediction error was 127.94 ppm, corresponding to a relative prediction error of 1.95%. This close agreement provides preliminary support for the predictive performance of the model within the investigated experimental domain. However, because the selected condition was located at or near the boundary of the design space, this validation does not establish the presence of a true internal optimum.

### 3.3. Characterization of Turmeric Emulsion and Encapsulated Powder

#### 3.3.1. Emulsion Properties

High-pressure homogenization is a widely used technology in the formation of nanoemulsion and nanoparticle systems. In this study, bioactive compounds extracted with deep eutectic solvents were pre-emulsified with a double-layer coating material (whey protein and maltodextrin) using a high-speed homogenization method. Subsequently, these coarse emulsions were subjected to high-pressure homogenization to reduce the particle size. However, during laboratory studies, the increase in system temperature along with the emulsification process applied under high pressure caused an unexpected increase in particle size. This situation can be explained by the fact that the increase in temperature reduces the effectiveness of surfactants and leads to droplet aggregation, as highlighted by Tadros et al. in the literature [29]. Therefore, it was decided to carry out the emulsification with a single-pass system in this study. Also, since the ultimate goal of the study was to produce the powder form product obtained by spray drying this intermediate system, not the emulsion form, the size of the emulsion alone was not considered a critical parameter. The particle size of 3.15 µm obtained in the samples demonstrated sufficient performance in terms of solubility, active substance retention, and functionality of the product after drying. Therefore, when evaluated in terms of the suitability of the product for its intended use, the inability to reach the nano size does not constitute a limiting factor from a scientific and technological perspective.

The emulsion properties of turmeric extracts produced by high-pressure homogenization method are given in Table S3. Zeta potential, particle size and polydispersity index (PDI) are among the most important parameters affecting emulsion stability and dispersion behavior. The mean particle size, PDI, and zeta potential were 3.15 ± 0.09 µm, 0.282 ± 0.068, and +9.38 ± 0.22 mV, respectively. Thus, the system was a micrometer-scale emulsion rather than a nanoemulsion. Because the primary objective was to produce an encapsulated powder by spray drying, the emulsion was used as an intermediate feed system. However, the suitability of this particle size for solubility, active-compound retention, or biological functionality cannot be established solely from the particle-size measurement. Zeta potential provides information about the surface charge of dispersed droplets and the possible contribution of electrostatic repulsion to dispersion stability. The relatively low absolute zeta potential measured in this study (+9.38 mV) does not indicate strong electrostatic stabilization and is insufficient to conclude that the droplets were resistant to aggregation. Steric and interfacial effects associated with whey protein isolate, Tween 80, maltodextrin, and modified starch may also have influenced the initial dispersion characteristics; however, these mechanisms were not directly investigated. Furthermore, storage stability, creaming or sedimentation, and time-dependent changes in particle size were not evaluated. Therefore, the reported measurements represent only the initial properties of the freshly prepared emulsion and do not demonstrate long-term colloidal stability [30].

PDI is a fundamental parameter that expresses the distribution width of particle sizes in a sample. This value, which varies numerically between 0.0 and 1.0, indicates that the system exhibits a monodisperse (uniform size) structure as it approaches 0.0, and a polydisperse (multiple size) structure as it approaches 1.0 [31]. When Table S3 is examined, it is seen that the PDI value of the turmeric emulsion produced by the high-pressure homogenization method is 0.282. This value reveals that the particles in the emulsion system show a relatively homogeneous distribution. So, the size distribution is concentrated in a narrow range. Although the homogenization process under high pressure was limited in reducing the particle size to the desired nano level, the low PDI value obtained has met the size homogeneity requirement, which is critical especially for the spray drying process. This situation shows that the system forms a suitable basis for the production of functional powder forms, which will directly and positively affect the product quality after drying.

#### 3.3.2. Physicochemical Properties of Encapsulated Turmeric Extract Powder

The results regarding the drying efficiency, moisture content, and water activity values of encapsulated turmeric extract powder are given in Table S4. Table S4 shows that the drying efficiency of the encapsulated powders obtained by the high-pressure homogenization method was 83.55%. The moisture content of the encapsulated turmeric extract powder was determined to be 3.03%, while the water activity value was determined to be 0.125.

#### 3.3.3. Flowability, Wettability, and Solubility Properties

The findings regarding the wettability and solubility properties of double-encapsulated turmeric extract powder produced by the spray-drying method are presented in Table S5. Wettability, which describes the dispersion of the powder by absorbing the liquid when it comes into contact with water, directly affects the rate of reconstitution of the product in water. As a result of the analyses, it was observed that the turmeric extract powders were completely wetted (homogeneously dispersed) in 397.5 s after being added to water. This time reflects the effectiveness of the interactions between the encapsulation material, the particle surface and the water. In addition, the solubility value, which indicates the water dissolution capacity of the powder product, was determined to be 78.34%. This rate shows that both the encapsulation process and the carrier system used were successful in terms of solubility performance. These results reveal that the produced powder has high potential in terms of ease of reuse and applicability in functional foods.

Powdered foods are more resistant to spoilage due to their low water content and, consequently, have a longer shelf life compared to fresh samples. Bulk density, an important criterion in the storage and packaging of powdered foods, affects the stability and shelf life of the products. In powders with low bulk density, the presence of more air between voids increases the likelihood of oxidation, thus reducing the storage stability of the powders. In this study, the bulk density, compressed bulk density, and flowability (Carr Index) values of encapsulated turmeric extract powders were examined. The bulk density of turmeric extract powders was found to be 324.15, while the compressed bulk density was determined to be 418.70 (Table S5).

The flowability of a powdered food is defined as the movement of a bulk of particles by rubbing against the surrounding particles or the walls of a container [32]. According to the Carr Index classification based on the bulk and tapped densities of powder products [33], CI values below 15 indicate very good flowability, values between 15 and 20 indicate good flowability, values between 20 and 35 indicate poor flowability, values between 35 and 45 indicate very poor flowability, and values above 45 indicate extremely poor flowability. The CI values of the encapsulated turmeric extract powder obtained in the study were determined as 22.58. The encapsulated turmeric extract powder had a CI value of 22.58%, corresponding to poor flowability according to the applied classification. Future studies should investigate agglomeration or other particle-engineering approaches to improve the flow properties of the powder.

#### 3.3.4. Curcuminoid Retention and Encapsulation Efficiency

The total and surface curcuminoid contents of encapsulated turmeric extract powders are presented in Table S6. When the results are examined, the total contents of bisdemethoxycurcumin, desmethoxy curcumin, and curcumin in the encapsulated powders were determined to be 123.71 ppm, 64.49 ppm, and 73.39 ppm, respectively, while the surface contents were found to be 3.04 ppm, 7.39 ppm, and 22.97 ppm. Based on these findings, it is concluded that bisdemethoxycurcumin and desmethoxy curcumin compounds are encapsulated in higher proportions compared to curcumin during the encapsulation process.

On the other hand, encapsulation efficiency is considered one of the most critical parameters in evaluating encapsulation success, indicating the extent to which bioactive compounds can be retained within the capsule structure. This parameter is directly affected by many factors such as the chemical and physical properties of the selected wall material, the stability of the emulsion formed, and the spray-drying conditions applied [34,35]. Encapsulation efficiency is generally calculated by comparing the total bioactive compound encapsulated with the amount free on the surface. In other words, as the amount of compound free on the surface decreases, the effective encapsulation success increases. In this context, in the analyses performed, the curcuminoid-based encapsulation efficiency of turmeric powder encapsulated with a double-layer system was determined as 87.33%. This high value shows both that the encapsulation capacity of the carrier system used is strong and that the emulsion stability and drying process are successfully managed. In addition, this result is quite valuable in terms of showing that an effective encapsulation environment is provided in protecting the bioactive components of turmeric against oxidation, heat and light.

### 3.4. Behavior of Encapsulated Turmeric Extract Powder Under Separately Simulated Gastric and Intestinal Conditions

The behavior of the encapsulated turmeric extract powder was evaluated under separately simulated gastric and intestinal conditions. The percentages of curcuminoids detected after the gastric and intestinal treatments were 31.24% and 31.10%, respectively (Table S7), indicating similar curcuminoid recovery under the two independently simulated conditions. Because the gastric and intestinal incubations were not performed sequentially, these values should not be interpreted as changes occurring during gastrointestinal transit or as evidence of targeted intestinal release.

In contrast to the similar curcuminoid recovery values, the measured ABTS radical-scavenging activity was higher after the simulated intestinal treatment and reached 81.57%. Previous studies on curcumin-loaded emulsion systems have reported that intestinal conditions can affect curcumin dispersion and its incorporation into mixed structures. Chen et al. [6] reported that bile salts and intestinal digestion conditions promoted the release and stabilization of curcumin encapsulated within nanoemulsion systems. Likewise, Ban et al. [36] observed improved curcumin bioaccessibility during simulated gastrointestinal digestion, which was associated with mixed micellar structures formed in the intestinal environment. However, these studies employed gastrointestinal models that are not directly equivalent to the independently conducted treatments used in the present study. Therefore, the current results provide only a comparison of the powder’s behavior under the two separately simulated conditions.

The similarity between the detected total phenolic content and curcuminoid recovery suggests that curcuminoids may constitute an important fraction of the measurable phenolic compounds. Nevertheless, the high antioxidant activity measured after the simulated intestinal treatment cannot be attributed solely to curcuminoids. Residual components of the thymol/lactic acid-based HDES, particularly thymol, may have contributed to the measured antioxidant activity because thymol possesses intrinsic antioxidant properties. However, thymol and lactic acid residues were not quantified in the final encapsulated powder. Their presence and relative contribution to the measured antioxidant activity therefore remain unknown.

A limitation of this study is that the gastric and intestinal treatments were performed independently rather than sequentially. In addition, free curcumin, non-encapsulated HDES extract, a physical mixture of the formulation components, a blank carrier system, and a conventional solvent extract were not included as comparative controls. Consequently, the results cannot demonstrate targeted release, directly represent gastrointestinal bioaccessibility, or establish that encapsulation enhanced curcuminoid recovery relative to non-encapsulated or conventionally extracted samples. The absence of residue quantification also prevents assessment of consumer exposure to thymol and lactic acid and precludes conclusions regarding the suitability of the powder for food or supplement applications.

### 3.5. Effect of HDES-Based Encapsulated Turmeric Extract Powders on Prostate and Colon Cancer Cells

**Figure 4:**
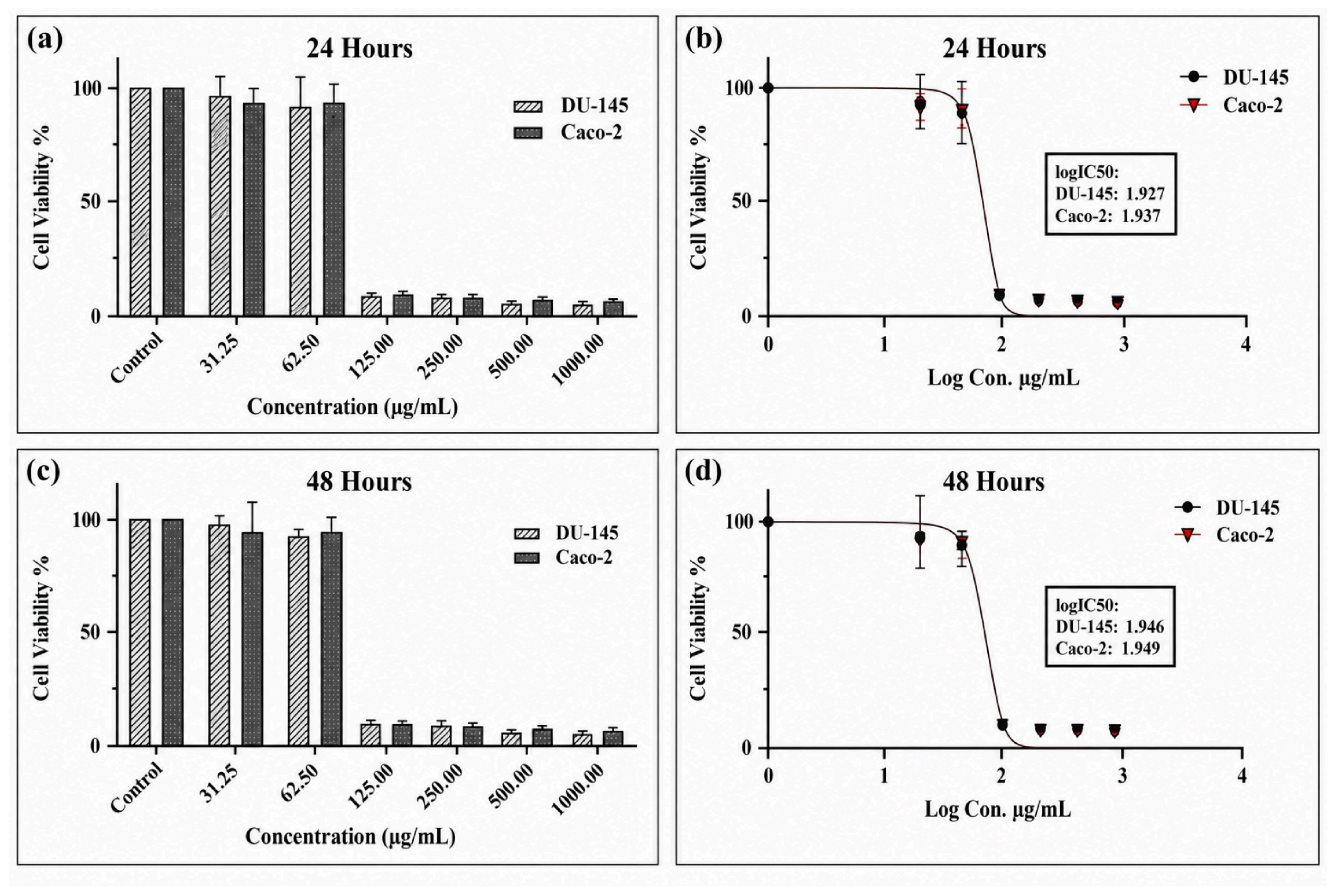
Cell viability of DU-145 (prostate cancer) and Caco-2 (colon cancer) cells after 24 and 48 h exposure to turmeric extract powders. (**a**) Cell viability after 24 h exposure. (**b**) Dose–response curves and corresponding logIC_50_ values after 24 h exposure. (**c**) Cell viability after 48 h exposure. (**d**) Dose–response curves and corresponding logIC_50_ values after 48 h exposure.

In this study, the cytotoxic potential of HDES-based turmeric extract powder particles (produced under optimum conditions) was investigated on DU-145 (human prostate adenocarcinoma) and Caco-2 (human colorectal adenocarcinoma) cell lines using the MTT test. Experimental applications included incubation periods of 24 and 48 h, while different concentrations (31.25–1000 µg/mL) of turmeric particles were applied to both cell lines. The percentage of cell viability was calculated. Furthermore, IC_50_ and logIC_50_ values were determined, whereas the results were confirmed with dose-dependent inhibition curves. After 24 h of incubation, the IC_50_ value was determined as 84.62 µg/mL in DU-145 cells and 86.52 µg/mL in Caco-2 cells (Table 4). These values reveal that turmeric extract particles exhibit a similar level of antiproliferative effect on both cell types during short-term exposure. LogIC_50_ values calculated using logarithmic conversion also support these findings (DU-145: 1.927, Caco-2: 1.937). After 48 h of incubation, the IC_50_ values were determined to be 88.29 µg/mL for DU-145 and 88.98 µg/mL for Caco-2. This indicates that longer exposure does not significantly increase toxicity. The effects may stabilize over time. LogIC_50_ values for this period were also determined to be 1.946 (DU-145) and 1.949 (Caco-2), respectively. Graphical analyses (Figure 4) showed a significant dose-dependent loss of viability in both cell lines. Particularly at doses of 125 µg/mL and above, cell viability dropped below 10%, indicating that turmeric particles have an inhibitory effect on cell proliferation.

Recent studies provide qualitative context for the concentration-dependent reduction in MTT metabolic activity observed in the present study. Boccellino et al. [37] exposed DU-145 cells to purified curcumin and reported a dose-dependent reduction in MTT viability after 24 h, with approximately 80% cell death at 55.2 µg/mL. Although the same cell line, exposure period, and MTT endpoint were used, direct comparison with the present IC_50_ values is not appropriate because our reported concentrations represent the mass of the complete encapsulated powder rather than the concentration of purified curcumin. Al-Duais et al. [38] evaluated nanocurcumin and nanocurcumin-containing composites in Caco-2 cells using an MTT assay after 24 h of exposure and reported concentration-dependent reductions in cell viability. However, their formulations consisted of gum Arabic-based nanocurcumin, selenium nanoparticles, and fucoidan-containing composites and differed substantially from the whey protein isolate–maltodextrin encapsulated HDES-derived powder investigated here. Their study also included cisplatin as a positive control and employed complementary DNA damage and ultrastructural analyses. These studies demonstrate that curcumin and curcumin-containing formulations can affect MTT metabolic activity in DU-145 and Caco-2 cells, but they do not establish that curcumin alone was responsible for the response observed in the present study. The absence of a blank carrier control, a pharmacological positive control, quantitative HDES residue analysis, and complementary mechanistic assays prevents attribution of the measured response specifically to curcuminoids and limits direct comparison with previous formulations.

Eventually, the HDES-derived curcuminoid-rich powder caused a concentration-dependent reduction in MTT metabolic activity in both DU-145 and Caco-2 cells. However, because pharmacological positive control and non-cancerous cell lines were not included, the potency and selectivity of the powder could not be established. Moreover, the MTT assay alone cannot distinguish among reduced metabolic activity, inhibition of cell proliferation, and induction of cell death. Therefore, these findings should be regarded as preliminary observations requiring confirmation using appropriate positive controls, non-cancerous cell models, and complementary mechanistic assays.

### 3.6. Limitations and Future Perspectives

This study has several limitations. The selected thymol/lactic acid mixture was characterized only by macroscopic appearance, viscosity, density, and apparent pH. Since DSC, FTIR, and NMR analyses were not performed, eutectic formation and the proposed intermolecular interactions were not independently confirmed. In addition, the safety of the selected mixture was not experimentally assessed, and thymol and lactic acid residues were not quantified in the final powder. These limitations prevent conclusions regarding HDES exposure and the suitability of the formulation for functional-food or supplement applications. Future studies should include thermal and spectroscopic characterization, quantitative residue analysis, and formulation-specific toxicological assessment. In addition, the colloidal measurements were performed on the freshly prepared emulsion, and no storage-stability study or time-dependent particle-size analysis was conducted. Therefore, long-term resistance to aggregation, creaming, or sedimentation could not be established.

The simulated gastric and intestinal treatments were conducted independently rather than sequentially. Moreover, free curcumin, non-encapsulated HDES extract, a physical mixture, a blank carrier system, and a conventional solvent extract were not included as comparative controls. Therefore, the findings cannot be interpreted as evidence of targeted intestinal release, as a direct measure of gastrointestinal bioaccessibility, or as proof that encapsulation enhanced curcuminoid recovery. In addition, thymol and lactic acid residues were not quantified in the final powder, preventing determination of their contribution to the measured antioxidant activity.

The cell-based evaluation was limited to the MTT assay in two cancer cell lines and did not include a pharmacological positive control, a blank carrier control, non-cancerous cell models, or complementary mechanistic assays. Consequently, the observed reduction in metabolic activity cannot be attributed specifically to curcuminoids or interpreted as evidence of selective antiproliferative or anticancer activity. Future studies should employ sequential gastrointestinal digestion, quantify residual HDES components and evaluate formulation safety. Cell-based studies should include appropriate positive and formulation controls, non-cancerous cells, and complementary assays addressing proliferation, apoptosis and cellular uptake. Recent advances in curcumin delivery also emphasize the use of standardized formulations and physiologically relevant three-dimensional cell models, which might provide a more reliable assessment of biological activity.

## 4. Conclusions

This study demonstrated the feasibility of combining thymol/lactic acid-based HDES extraction with double-layer emulsification and spray-drying to produce a curcuminoid-rich turmeric powder. Among the investigated HDES systems, the thymol/lactic acid system provided the highest total curcuminoid concentration in the analyzed extract solution. The resulting encapsulated powder had a particle size of 3.15 µm, a PDI of 0.282, and a total curcuminoid-based encapsulation efficiency of 87.33%.

Under separately simulated gastric and intestinal conditions, similar curcuminoid recovery values were obtained, whereas higher ABTS radical-scavenging activity was measured after the simulated intestinal treatment. Because these treatments were performed independently rather than sequentially, the results cannot be interpreted as evidence of targeted intestinal release or as a direct measure of gastrointestinal bioaccessibility. Furthermore, thymol and lactic acid residues were not quantified in the final powder. Therefore, a possible contribution of residual HDES components to the measured antioxidant activity cannot be excluded.

The encapsulated powder also caused a concentration-dependent reduction in MTT metabolic activity in DU-145 and Caco-2 cells. However, the absence of a pharmacological positive control, non-cancerous cell models, and complementary mechanistic assays limits conclusions regarding its antiproliferative potency, cytotoxic mechanism, and selectivity. Consequently, these findings should be considered preliminary.

The results support the technical feasibility of the proposed extraction and encapsulation approach. However, the formation and molecular interactions of the selected thymol/lactic acid system were not confirmed by thermal or spectroscopic analyses. Further studies should include DSC and spectroscopic characterization, sequential gastrointestinal digestion, quantitative analysis of residual HDES components, safety assessment, appropriate cell-assay controls, and complementary biological assays before conclusions regarding intestinal bioaccessibility, functional-food applications, or anticancer potential can be drawn.

## Figures and Tables

**Table 1:** HPLC-determined concentrations of individual and total curcuminoids in the analyzed HDES-based turmeric extract solutions.

No	Demethoxycurcumin (ppm)	Bisdemethoxycurcumin (ppm)	Curcumin (ppm)	Total Curcuminoid * (ppm)
1	1949.31	1095.36	1042.12	4086.79
2	1379.93	943.47	1004.93	3328.33
3	1295.50	1051.67	1166.04	3513.21
4	1303.88	941.23	972.68	3217.79
5	1437.41	1139.14	1289.20	3865.75
6	1528.84	1107.84	1240.14	3876.82
7	1464.46	1200.87	1393.31	4058.64
8	1348.17	1116.78	1282.23	3747.17
9	1314.76	1073.16	1259.10	3647.02
10	922.23	737.42	759.63	2419.28
11	1004.76	749.02	801.96	2555.75
12	1560.00	1168.65	1226.50	3955.15
13	2489.74	1561.18	1588.55	5639.46
14	2272.21	1430.97	1468.03	5171.22
15	2056.43	1442.79	1498.00	4997.22
16	1860.19	1373.75	1460.88	4694.82
17	1373.63	1138.87	1288.65	3801.15
18	1561.17	1269.78	1423.62	4254.57
19	1511.44	1206.87	1349.38	4067.69
20	1640.61	1269.12	1420.61	4330.34
21	738.61	961.80	1308.72	3009.14
22	1554.75	1240.36	1400.64	4195.74
23	1573.45	1177.45	1301.08	4051.97
24	1569.46	1182.68	1301.08	4053.22
25	1165.03	954.43	1025.86	3145.32
26	762.02	625.26	699.66	2086.94
27	642.22	940.64	1301.04	2883.91
28	2672.72	1701.01	1756.63	6130.36
29	2521.31	1640.64	1682.45	5844.40
30	2744.78	1777.24	1827.58	6349.60

* Total curcuminoid concentration was calculated as the sum of demethoxycurcumin, bisdemethoxycurcumin, and curcumin. The reported ppm values refer to the final analytical solutions submitted to HPLC and not to dry turmeric weight.

**Table 2:** Box–Behnken process parameters and total curcuminoid concentrations determined in the analyzed extract solutions.

No	A: Solid Mass (g)	B: Immersion Time (min)	C: Washing Time (min)	Y_TC_: Total Curcuminoid Concentration (ppm)
1	1	20	30	2349.96
2	0.5	20	40	3017.00
3	1.5	20	20	2680.21
4	1	10	20	89.80
5	0.5	10	30	676.96
6	1	20	30	2108.60
7	0.5	30	30	270.12
8	0.5	20	20	720.13
9	1	30	40	4708.56
10	1	20	30	2250.21
11	1.5	10	30	1102.30
12	1	20	30	1900.23
13	1	30	20	2700.10
14	1	20	30	2300.32
15	1.5	20	40	6534.19
16	1.5	30	30	4296.88
17	1	10	40	4510.19

**Table 3:** Analysis of variance (ANOVA) for the quadratic model fitted to the total curcuminoid concentration in the analyzed extract solution.

Source	Sum of Squares	Degrees of Freedom	Mean Square	F-Value	*p*-Value	Significance
Model	4.835 × 10^7^	9	5.372 × 10^6^	137.88	<0.0001	Significant
A	1.232 × 10^7^	1	1.232 × 10^7^	316.31	<0.0001	
B	3.915 × 10^6^	1	3.915 × 10^6^	100.48	<0.0001	
C	1.978 × 10^7^	1	1.978 × 10^7^	507.71	<0.0001	
AB	3.243 × 10^6^	1	3.243 × 10^6^	83.22	<0.0001	
AC	6.061 × 10^5^	1	6.061 × 10^5^	15.56	0.0056	
BC	1.454 × 10^6^	1	1.454 × 10^6^	37.33	0.0005	
A^2^	1.361 × 10^5^	1	1.361 × 10^5^	3.49	0.1038	Not significant
B^2^	7.269 × 10^5^	1	7.269 × 10^5^	18.66	0.0035	
C^2^	6.430 × 10^6^	1	6.430 × 10^6^	165.04	<0.0001	
Error	2.727 × 10^5^	7	38,961.65			
Lack-of-fit	1.411 × 10^5^	3	47,028.98	1.43	0.3585	Not significant
Pure error	1.316 × 10^5^	4	32,911.15			
Cor total	4.862 × 10^7^	16				

**Table 4:** logIC_50_ and IC_50_ values obtained after 24 and 48 h treatment of turmeric extract powders on DU-145 and Caco-2 cells.

24 h				48 h			
DU-145		Caco-2		DU-145		Caco-2	
LogIC_50_:	1.927	LogIC_50_:	1.937	LogIC_50_:	1.946	LogIC_50_:	1.949
IC_50_:	84.62	IC_50_:	86.52	IC_50_:	88.29	IC_50_:	88.98

## Data Availability

The original contributions presented in the study are included in the article, further inquiries can be directed to the corresponding author.

## Supplementary Materials

The following supporting information can be downloaded at: https://www.mdpi.com/article/10.3390/foods15152601/s1, Table S1: Components and molar ratios of the prepared hydrophobic solvent mixtures; Table S2: Selected physicochemical properties of the prepared hydrophobic solvent mixtures; Table S3: Initial particle-size distribution and zeta potential of the freshly prepared HDES-based turmeric emulsion; Table S4: Physicochemical properties of the spray-dried encapsulated turmeric extract powder; Table S5: Bulk density, tapped density, Carr Index, wettability, and solubility of the encapsulated turmeric extract powder; Table S6: Total and surface curcuminoid contents of the encapsulated turmeric extract powder; Table S7: Recovery of bioactive compounds under independently simulated gastric and intestinal conditions; Figure S1: Representative HPLC chromatogram of the curcumin standard; Figure S2: HPLC calibration curves for bisdemethoxycurcumin, demethoxycurcumin, and curcumin; Figure S3: Overlay of the HPLC chromatograms of the Experiment 26 sample before and after standard addition; Figure S4: Numerical desirability ramp plot illustrating the selected factor levels for maximizing the total curcuminoid concentration within the investigated experimental domain.
