# Supplementary material for: Hydrophobic Deep Eutectic Solvent-Derived Curcuminoids Loaded into a Double-Layer Colloidal Delivery System: Physicochemical Properties and In Vitro Biological Activity
Source: Foods. 2026 Jul 24;15(15):2601. doi: 10.3390/foods15152601 (PMC13464657; doi:10.3390/foods15152601)
Supplement: Supplementary file 1 [file foods-15-02601-s001.zip › foods-4441800-supplementary.pdf]

Table S1. Hydrophobic deep eutectic solvent mixture components and molar ratios.

No	HBA	HBD	Molar ratio
1	Menthol	Oleic acid	1:1
2	Menthol	Oleic acid	1:2
3	Menthol	Oleic acid	2:1
4	Menthol	Capric acid	1:1
5	Menthol	Capric acid	1:2
6	Menthol	Capric acid	2:1
7	Menthol	Caprylic acid	1:1
8	Menthol	Caprylic acid	1:2
9	Menthol	Caprylic acid	2:1
10	Menthol	Pyruvic acid	1:1
11	Menthol	Pyruvic acid	1:2
12	Menthol	Pyruvic acid	2:1
13	Menthol	Lactic acid	1:1
14	Menthol	Lactic acid	1:2
15	Menthol	Lactic acid	2:1
16	Thymol	Oleic acid	1:1
17	Thymol	Oleic acid	1:2
18	Thymol	Oleic acid	2:1
19	Thymol	Capric acid	1:1
20	Thymol	Capric acid	1:2
21	Thymol	Capric acid	2:1
22	Thymol	Caprylic acid	1:1
23	Thymol	Caprylic acid	1:2
24	Thymol	Caprylic acid	2:1
25	Thymol	Pyruvic acid	1:1
26	Thymol	Pyruvic acid	1:2
27	Thymol	Pyruvic acid	2:1
28	Thymol	Lactic acid	1:1
29	Thymol	Lactic acid	1:2
30	Thymol	Lactic acid	2:1

Table S2. Some physicochemical properties of the prepared HDESs*.

No	Viscosity (mPa.s)	Density ($g.cm^{-3}$)	Apparent pH**
1	38.07	0.8152	1.54
2	36.87	0.8325	0.30
3	34.47	0.8555	1.16
4	18.58	0.8618	0.45
5	14.67	0.8748	0.18
6	26.38	0.8671	1.51
7	14.18	0.8843	0.11
8	20.36	0.8812	0.77
9	20.68	0.8676	0.89
10	22.14	0.8461	0.55
11	27.35	0.8081	0.45
12	19.23	0.6769	0.15
13	37.48	1.0984	0.33
14	32.67	1.1227	0.30
15	57.46	1.0107	0.49
16	27.29	0.8717	0.32
17	29.89	0.8701	0.40
18	24.88	0.8842	0.32
19	14.68	0.9125	0.21
20	15.88	1.0010	0.28
21	17.29	0.8112	0.30
22	11.10	0.9315	0.37
23	35.25	1.0877	0.36
24	36.15	1.1225	0.38
25	12.32	0.8214	0.09
26	15.65	0.7115	0.15
27	15.36	0.8014	0.20
28	26.98	1.168	0.40
29	26.98	1.1894	0.43
30	27.88	1.0551	0.37

*Values represent single measurements; therefore, standard deviations and inferential statistical comparisons are not reported.
**Apparent pH values are reported as comparative measurements because the systems were predominantly non-aqueous.

Table S3. Initial particle size distribution and zeta potential of the freshly prepared HDES-based turmeric emulsion produced by high-pressure homogenization*.

Zeta potential (mV)	Particle size (µm)	PDI
9.38±0.22	3.15±0.09	0.282±0.068

*Values are expressed as mean ± standard deviation (n = 3).

Table S4. Physicochemical properties of spray-dried encapsulated turmeric extract powder*.

Drying yield (%)	Moisture content (%)	Water activity
83.55±3.55	3.03±0.30	0.125±0.004

*Values are expressed as mean ± standard deviation (n = 3).

Table S5. Bulk density, tapped density, flowability, wettability, and solubility properties of the encapsulated turmeric extract powder*.

Bulk density (kg/m^3)	Tapped density (kg/m^3)	Carr index	Wettability (s)	Solubility (%)
324.15±1.29	418.70±1.66	22.58±0.65	397.5±13.3	78.34±0.77

*Values are expressed as mean ± standard deviation (n = 3).

Table S6. Total and surface curcuminoid contents of the encapsulated turmeric extract powder produced by high-pressure homogenization and spray drying*.

Sample	Bisdemethoxycurcumin (ppm)	Demethoxycurcumin (ppm)	Curcumin (ppm)	Total curcuminoids (ppm)
ETP** total	123.71±0.82	64.49±0.44	73.39±1.75	263.59±2.13
ETP surface	3.04±1.14	7.39±0.30	22.97±0.36	33.39±0.47

*Values are expressed as mean ± standard deviation (n = 3).
**ETP: Encapsulated turmeric extract powder.

Table S7. *In vitro* release behavior of bioactive compounds from encapsulated turmeric powder produced by high-pressure homogenization during separate gastric and intestinal treatments*.

Sample	Antioxidant activity (%)	Total phenolic content (%)	Total curcuminoids (%)
ETP gastric phase	13.14±0.12	25.94±1.00	31.24±0.54
ETP intestinal phase	81.57±1.12	31.21±1.10	31.10±0.84

*Values are expressed as mean ± standard deviation (n = 3).

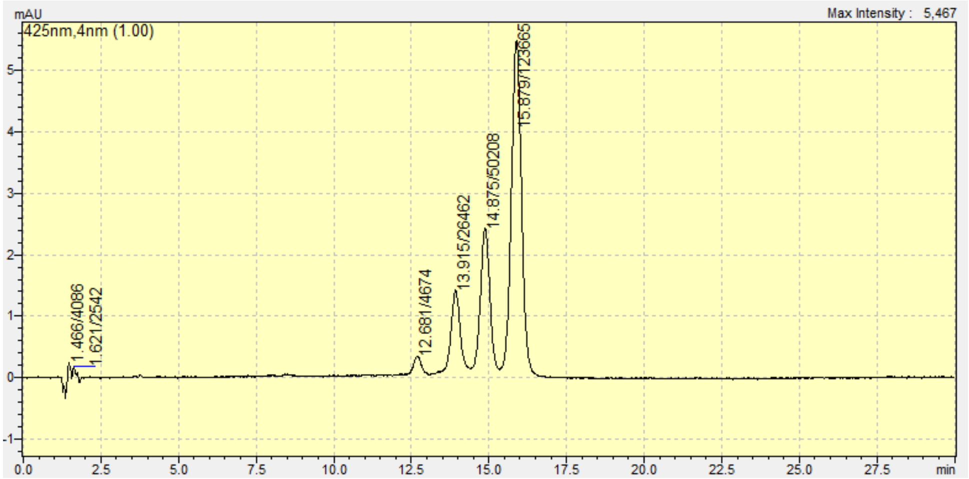

Figure S1. Representative HPLC chromatogram of the curcumin standard recorded at 425 nm. Retention time is shown in minutes, and detector response is expressed in mAU.

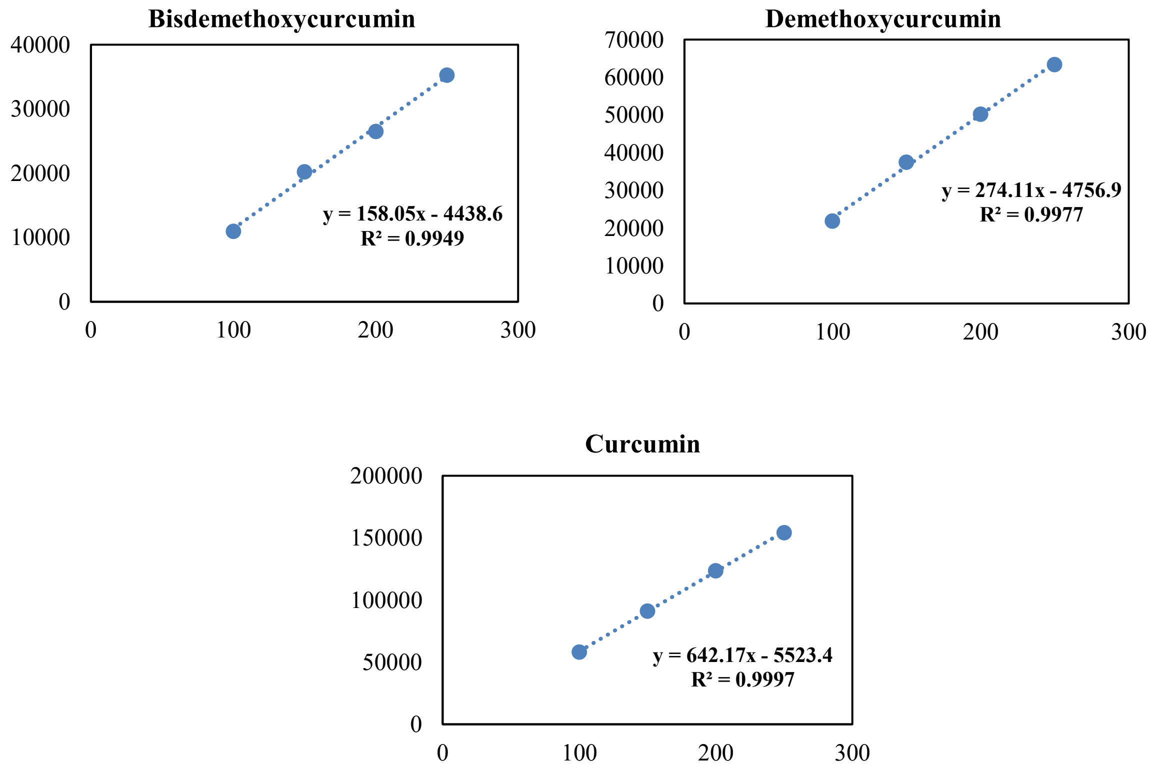

Figure S2. HPLC calibration curves for bisdemethoxycurcumin, demethoxycurcumin, and curcumin.

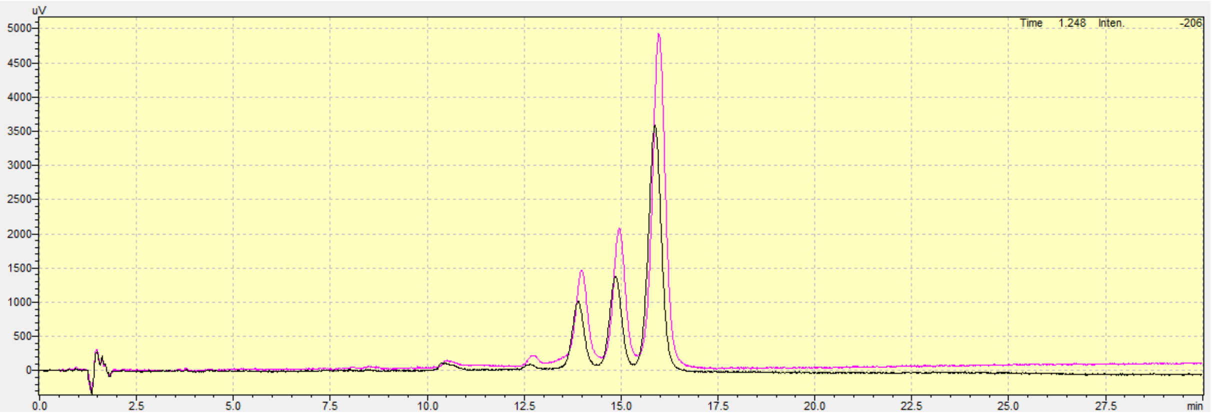

Figure S3. Overlay of the HPLC chromatograms of the sample from Experiment 26 before (black) and after standard addition (pink).

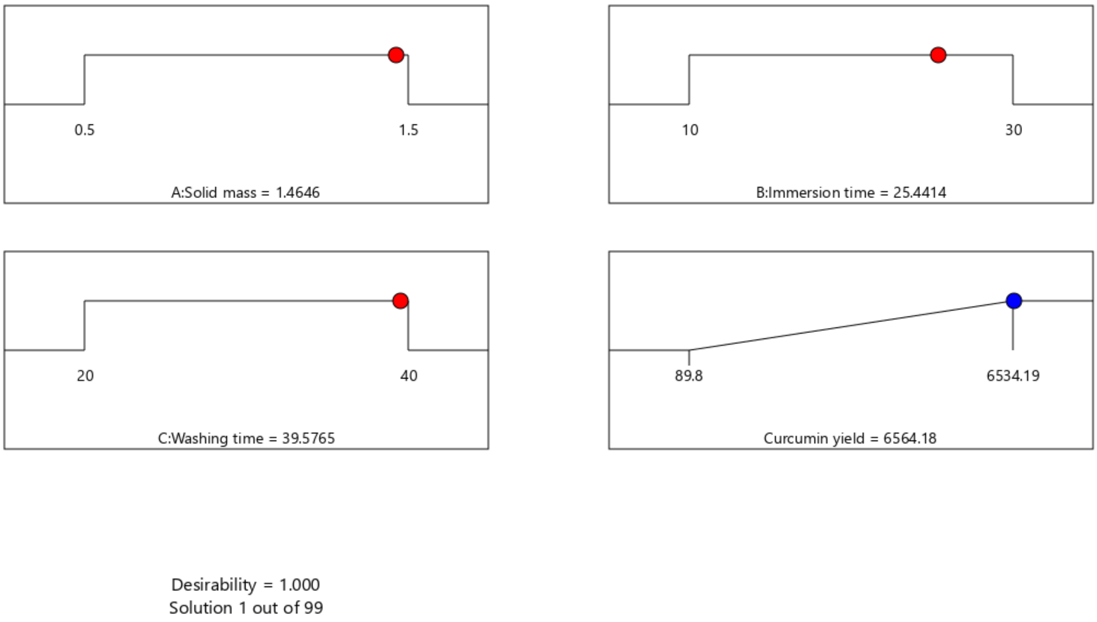

Figure S4. Numerical desirability ramp plot illustrating the optimum levels of solid mass, immersion time and washing time for maximum total curcuminoid concentration in the analyzed extract solution.
